# Neuroendocrine Tumors: Treatment and Management

**DOI:** 10.3390/cancers14164048

**Published:** 2022-08-22

**Authors:** Alessio Imperiale

**Affiliations:** 1 Nuclear Medicine and Molecular Imaging Institut de Cancérologie Strasbourg Europe (ICANS), 17 Rue Albert Calmette, 67033 Strasbourg, France; a.imperiale@icans.eu; Tel.: +33-3-68-76-74-48; Fax: +33-3-68-76-72-56; 2 University Hospitals of Strasbourg, Faculty of Medicine, University of Strasbourg, 67000 Strasbourg, France; 3 Molecular Imaging—DRHIM IPHC, UMR7178, CNRS/Unistra, 67000 Strasbourg, France

This Topical Collection consists of a series of articles presented by a panel of internationally recognized experts and compiles several clinical accomplishments in the field of neuroendocrine tumors (NETs). It consists of 15 papers, both original articles and reviews, covering currently debated epidemiologic, diagnostic, and therapeutic issues of particular interest to physicians involved in the management of patients with this pathological condition.

NETs are rare and heterogeneous epithelial neoplasms with neuroendocrine differentiation commonly originating from the gastroenteropancreatic (GEP) system and lung. NET patients are often asymptomatic or present with incidental findings on imaging studies. Usually, clinical symptoms are associated with advanced disease and the presence of systemic metastatic spread. In recent years, considerable progress has been made in the therapeutic strategy for NETs, underlining that a multidisciplinary approach remains essential in the therapeutic discussion of patients with metastatic disease. The only curative treatment for patients with NETs is surgery, including resection of the primary tumor. However, considering their long-term survival, patients are likely to receive multiple treatments, although the optimal therapeutic strategy remains to be defined.

Takayanagi et al. [1] provided an overall update of the epidemiology, diagnosis, and clinical biomarkers in GEP-NETs. Both the incidence and prevalence of GEP-NETs has been increasing worldwide over the last four decades, especially in the small intestine and rectum, probably due to the aging population and the recent advances in diagnostic techniques, including endoscopy and imaging investigations. Shah et al. [2] reported the results of a retrospective analysis of a national database investigating the incidence and survival of lung neuroendocrine neoplasms in the United States. Accordingly, overall survival and disease-specific survival trends are significantly related to tumor stage, histological type, but also age, race, marital status, and insurance type. It is noteworthy that, despite the increase in newly diagnosed cases of lung neuroendocrine neoplasms in recent decades, the incidence of small cells lung cancer (SCLC) is reducing, likely due to declining smoking habits.

Medical imaging plays a crucial role in assessing tumor locoregional extension and metastatic spread, particularly in the liver. Accordingly, a variable combination of ultrasound (US), computed tomography (CT), magnetic resonance imaging (MRI), and multitracer positron emission tomography (PET) is typically adopted, considering the clinical context and both the strengths and limitations of each diagnostic modality. Treglia et al. [3] performed an umbrella review of 34 published meta-analyses to provide an evidence-based summary of the diagnostic performance, prognostic value, and impact on management and the safety of PET. The resulted findings support the use of functional imaging in NET patients with specific indications for each radiopharmaceutical, underlining the intimate relationship that exists between molecular imaging phenotype and tumor pathological features, potentially influencing the therapeutic approach.

Whenever feasible, surgery is proposed as first option for low-grade tumors, including those originating from small intestine (siNETs), pancreas (pNETs), and lung. Pasquer et al. [4] stressed the challenge of proposing a surgical resection without imposing short small bowel syndrome in patients with siNETs. The oncological benefits supported in the literature led to recent changes in the recommendations of academic societies. The management of pNETs is difficult due to their heterogeneity and the risks associated with pancreatic surgery. These patients should be managed in specialized, high-volume centers with multidisciplinary discussion. Innovative managements such as “watch and wait” strategies, parenchymal-sparing surgery, and minimally invasive approaches are emerging. As a result, de Ponthaud et al. [5] and Frey et al. [6] provide an update of the surgical management of pNETs and highlight selected key elements in view of the recent literature.

Approximately 80% of metastatic patients with GEP-NETs have liver metastases (LMs). Addeo et al. [7] retrospectively reviewed clinical data from a monocentric cohort of 51 consecutive patients who underwent the simultaneous resection of pNETs with LMs, underlining that a combined surgical procedure can be performed safely with acceptable morbidity and mortality. Well-differentiated pNETs had longer survival and might benefit the most from these combined surgeries. While a cure is the goal for localized tumors, it is rarely achievable in patients with metastatic disease. Thus, the preservation of quality of life, control of the secretory syndrome, treatment of complications, and prevention of toxicity of therapies are critical. Locoregional treatments will be proposed in cases where systemic treatment, such as cold somatostatin analogs, is ineffective. In selected patients with LMs for whom hepatic surgery is contraindicated, percutaneous or intra-arterial treatments are safe and effective options for achieving disease control. Cazzato et al. [8] provided a narrative review of the current knowledge on liver-directed therapy for LMs treatment, including both interventional radiology procedures and nuclear medicine options (90Y-Selective Internal Radiation Therapy (SIRT), Targeted Radionuclide Therapy) in NET patients.

Multiple therapeutic options are available for unresectable advanced disease or metastatic disease, including medical treatment with cold somatostatin analogs, peptide receptor radionuclide therapy (PRRT), chemotherapy, and molecule-targeted therapies, such as mammalian targets of rapamycin (mTOR) inhibitors and antiangiogenic agents. An update of the therapeutic management of well-differentiated, grade-3 NETs and neuroendocrine carcinomas is proposed by Pellat et al. [9,10], exploring future directions for their treatment and underlying the urgent need for more evidence to help define the best therapeutic strategy in these rare but aggressive diseases. Although radiotherapy represents a commonly used method of treating lung NETs, the available recommendations for lung NET radiotherapy are minor. This poses a serious problem when a patient needs to be directed to external radiotherapy. Bilski et al. [11] present the current knowledge on the use of radiotherapy in the treatment of lung NETs, providing a description of several clinical cases that could potentially help radiation oncologists to make the best and most personalized therapeutic decisions.

The use of in vivo functional diagnostic testing, such as nuclear medicine investigations, to explore the molecular mechanisms of an individual patient’s disease, is useful for a safe and effective therapeutic strategy. From a clinical perspective, the integration of diagnostics and therapeutics (theranostic) by in vivo molecular imaging represents a major opportunity to select appropriate treatment, monitor therapy, and determine prognosis. Targeted radionuclide therapy represents the most common example of patient-specific therapies based on the ‘image and treat’ approach”. In the context of NETs, this means the use of molecular vectors labeled either with diagnostic or therapeutic radionuclides. The only target currently used in clinical practice in NET patients is the somatostatin receptor, and one of the most interesting associations is ^68^Ga-DOTATATE and ^177^Lu-DOTATATE (somatostatin-analogues-based PRRT), with very promising results. Ahmadi Bidakhvidi et al. [12] provide a didactical review focusing on the basic principles and clinical applications of PRRT, discussing several PRRT-optimization strategies in patients with NETs. Moreover, Theiler et al. [13] report the results of PRRT in a cohort of elderly NET patients (>79 years old), resulting in a valid therapeutic option with similar toxicity and non-inferior survival compared to matched younger patients.

Toxicity and quality of life are important elements in the therapeutic decision and must be considered for each patient scheduled for PRRT. Thus, the early identification of non-responder patients could play a role in this by providing a personalized therapeutic approach and consequent reductions in iatrogenic toxicity. Radionuclide imaging such as ^18^F-FDG PET has potential value in the prognostic stratification of NET patients. NETs with increased ^18^F-FDG uptake are more aggressive and less favorable to long-term survival. Moreover, some evidence indicates that ^18^F-FDG PET plays a role in predicting response to ^177^Lu-PRRT monotherapy, allowing for the identification of patients with grade 1 and 2 metastatic NETs that might benefit from more intensive therapy protocols, including a combination of chemotherapy and PRRT. Overall, ^18^F-FDG PET appears interesting in disease prognostication, as it can influence the therapeutic strategy and the aggressiveness of patient management. Alevroudis et al. [14] provided a systematic review and meta-analysis evaluating the impact of ^18^F-FDG PET performed before PRRT in twelve studies, including 1492 NET patients of different origins. Therefore, ^18^F-FDG PET imaging prior to PRRT administration appears to be a useful tool in NET patients to predict tumor response and survival outcomes, and a negative ^18^F-FDG PET scan is associated with prolonged PFS and OS. By staying in the domain of PRRT efficacy prediction, Ruhwedel et al. [15] proposed the use of the so-called De Ritis ratio to identify patients with a favorable PRRT outcome in 125 NET patients, demonstrating that a high De Ritis ratio and high levels of Chromogranin A improved the prediction of progression-free survival after treatment.

In conclusion, we would like to thank all the authors for the papers they submitted to this Topical Collection, and all the reviewers for their careful and timely reviews, improving the quality of this Collection.
